# Her Heart's Mistake

**Published:** 1889-07-06

**Authors:** E. D. Cumming

**Affiliations:** Author of "An Ordeal by Silence"


### Her Heart's Mistake.

By E. D. Cumming, Author of "An Ordeal by Silence."

*(Continued from page 189.)*

### Chapter XII.—Cholera.

"But surely, Mr. Warren, you don't mean to imply that all clever women are fast?" said Mrs. Brent, putting down her work to join in an animated discussion which was in progress between her niece and the barrister.

Mr. Warren had been dining at the corner bungalow that evening, and the party had, as usual, adjourned to the verandah, where the Doctor slumbered in his chair, and the others read, worked, or talked.

"No, Mrs. Brent," said the gentleman, turning to lean his back against the railing, "I didn't say that. As I have been telling Miss Harrison, I don't use the expression 'fast' in its dictionary sense of changeable or inconstant, nor in its commoner aspersory significance."

"It usually means a reckless disregard for all the unwritten laws of society," said Mrs. Brent.

"The term is a very far-reaching one, and I use it in default of a better to describe women who excite remark by their unwillingness to be bound by the narrow limits which society prescribes for their freedom of action. You know far better than I do how short a distance a girl need go beyond them to be outlawed as 'fast,' and how often or how seldom the sentence is deserved. What I said was that every woman I have known who neglected the social boundaries was intellectually superior to the majority of those who remain contentedly within them."

"Then I should have correctly understood you to say that all fast women are clever?" Mrs. Brent was beginning to think that this young man had dangerously advanced opinions, and offered this challenge with the view of driving him into a corner.

"There you overstate my theory a little; but not much, premising that you give the word 'fast' the meaning I wish it to bear. I think that the narrowness of the sphere which society has defined as suitable and sufficient for women is most obvious, and most galling to those whose natural abilities are above the average. Those restrictions appear to them, not only needless but absurd, and they look beyond them to find scope for the energies and talents which are cramped and wasted in their own sphere."

"May I ask if you consider that the pursuits in which fast women indulge are evidences of superior ability—like driving tandem for instance?"

"I don't know that a fondness for driving two, or any number of horses, can be called direct evidence of intellectual power," said Warren with a smile. "But you name an offence, which I think throws a stronger light on the excessive rigour of the law, than upon the moral depravity of the nature which finds pleasure in committing it."

"Granting that the laws or the limits *are* unduly rigorous, do you see anything in such tastes to support your argument?"

Warren rubbed his chin thoughtfully and weighed the question before answering it.

"Yes," he said, after a pause. "I think I see the feeling of independence which is born of mental strength. If I may use your illustration, to be able to drive tandem requires more nerve and skill in a particular department than most women possess. Is it reprehensible that one who owns the necessary qualifications should wish to exercise them?"

"I think a wish to display their qualifications prompts them to do such things as a rule," replied Mrs. Brent, with some acrimony.

"Vanity is responsible for many of our actions," laughed Warren, "but I can't think that a love of showing off, as children call it, is the true secret of women's enterprise. The indulgence of such tastes as tandem-driving may or may not be commendable; but at the same time it argues disdain for these too circumscribed limits, and a capacity for taking wider views."

"Then are we to conclude that the cleverer a woman is, the farther beyond the limits she will go?" asked Mrs. Brent.

"No, her own intelligence will hold her in a leash. Please bear in mind that we are speaking of the harmlessly 'fast.'"

"Then do you adhere to your theory that harmlessly-fast people are generally clever?" asked Kate, who had been an interested listener since her aunt took up the argument.

"Undoubtedly; the higher the order of a woman's intellect, the more absurdly narrow does the scope defined by social usage appear to her. She looks out upon the world, and sees the greater work that men are doing; she knows herself competent to take a part in it, and breaks through the restraints against which other women of less ability only chafe restlessly. She feels herself above those occupations and interests which content her sisters of a still lower level of intelligence, who cannot, or do not, wish to look beyond their own cushioned circle."

"But don't you think that those who are contented do a greater amount of good and wield more extensive influence than those who break through the barriers?" enquired Kate.

"Always keeping in mind that we speak of the harmlessly fast, I think not. Clever women come more closely in touch with men, and make their influence more directly felt. They could do more were they not too often handicapped by bearing the undeserved reputation of 'fastness.'"

"Then it appears to me that were clever women to confine themselves to their own sphere they could do more than you think they do by going outside it," put in Mrs. Brent.

"If their own sphere gave them the necessary scope, undoubtedly they would; but it does not. I think it natural and right that a girl who is endowed with talents should develop a yearning, and seek a field in which to use them. The true solution of the difficulty is to widen that sphere."

"You think that it should be increased and enlarged, so as to take in the ground upon which women cannot now tread without the risk of being called fast?"

"Certainly; widen the sphere and they will be content to remain within it. Of course, no limits would be wide enough to satisfy those who, without talents or intelligence, break bounds in search of pleasure, and with no other aim. But those are the viciously fast, of whom I have nothing to say."

"Will it ever be widened?" asked Kate.

"The tendency of the age seems to be in that direction, but progress must necessarily be slow. I cannot fail to see that those best able to judge, by reason of their better understanding, consider their ground too restricted."

"I suppose clever people have more capacity for enjoyment in every shape than dull ones?"

"I think so; but I imagine that the dull people have a counter-balancing advantage, in that they are less susceptible to sorrow. Joy and sorrow come in fairly even proportions to most of us in this world. Still I do think that the intelligent man or woman reaps greater pleasure from life than the torpid individual," replied Warren, glad to drop the subject of "fast" women, which did not appear [illegible]o [illegible]e palatable to Mrs. Brent.

Thanks to his letter of introduction, he had had more opportunities of becoming acquainted with the Harrison household than with others in Meerut. The Doctor liked him, and frequently asked him to join them at dinner in an informal way. Kate had found in him a man who talked as no one else she knew could talk; he looked more deeply into things than most people, and as many of his views harmonised with her own, she often found herself absorbed in conversations with him of a kind she had never enjoyed with anyone of either sex before. Mrs. Brent did not care about him at all; she thought him didactic and revolutionary, as well as uncouth. He was not, in her estimation, a desirable companion for her niece, and rejoiced that Kate's affection for George Bairdsley, who was so much higher a class of man in every way, was a safeguard against Mr. Warren. The fact gave ease to her mind, but still she did not like to see the young barrister so much about the house. There was absolutely nothing in his demeanour towards Kate, nor in Kate's conduct with him, to which she could take exception as unbecoming a girl who was "engaged." But her dislike to Mr. Warren was steadily increasing, and she frequently caught herself weighing the advisability of taking her brother to task about asking him so often to the house. He never came without invitation by any chance, but the fact did nothing to eradicate the prejudice she had against him, and she always saw him take his departure from the bungalow with a sense of relief. She bade Mr. Warren a very stiff farewell when he left the house that night; and after telling her niece that she did not feel quite so well as usual, retired to her own room.

Her aunt was so often ailing and out of sorts when the weather was muggy and close as it was to-night that Kate felt no anxiety regarding her health, and went to bed soon afterwards, pondering over Mr. Warren's theory about fast women which opened up a vein of thought hitherto undiscovered. She was inclined to agree with his views herself, thinking that she could have suggested a cogent reason for clever women who sought men's society, rousing the vindictive feelings of their own sex in jealousy; she preferred not to say so before her aunt, but the next time the subject came up she would ask Mr. Warren if he did not think that that had much to do with it. It certainly was a real pleasure to converse with such a man; she was sure George would like him, and that Mr. Warren would pull well with George. Oh, if only he could come down from Simla; he had been away a long time now; how she would look forward to the day of his return when it should be fixed! But that was a long way away; very long; months and months; what a pity it was that the distance was so far, and that they couldn't find a shorter path than that tedious winding road George had described. And Kate's fancy was picturing a route direct from Simla to Meerut through snow-crowned heights and over bottomless abyss which would have made an engineer faint at its daring, when she fell asleep.

She was awakened, an hour or two later, by the excited murmuring of servants and bustle in the back verandah. Lamps were shining through the venetians, and Dr. Harrison's voice was audible giving rapid orders in Hindustani. She rose and dressed quickly, wondering what had gone wrong, for she saw the gharry come round from the stable and drive through the porch out on to the Mall without stopping, as though it had been despatched to fetch someone.

"Has anything happened, papa?" she enquired as she met him on her way through the drawing-room.

"Your aunt is very ill. Don't go in to her just now," he added as he saw her look towards the door.

The grave anxiety of her father's face warned Kate that this was not the time to ask questions; she returned to her own room and sat down behind the purdah to wait. From time to time she could hear moans from Mrs. Brent's room, but she knew that she would best serve the patient by obeying her father's instructions not to go into the room, and restrained her anxiety as best she could. Presently the sound of horses' hoofs and the rumble of wheels told her that whoever the gharry had been sent for was coming in all haste, and a moment later Dr. Anson's voice, speaking in low, eager tones to her father, could be heard outside. They went together into Mrs. Brent's room and remained there for a time that seemed an age to the anxious girl sitting alone in suspense. The groans grew louder and more frequent, till they made her blood run cold. She could not remain still in ignorance, and went out on to the verandah to try and learn the truth, but for half an hour she ascertained nothing. The bearer came out of Dr. Harrison's room, and told the coachman to take the gharry to the stable, for the doctor sahib would not want to go back. The chaukidar asked the bearer a question, and received an answer in Pushtu, which appeared to alarm him, but which Kate could not understand. She picked up a book and endeavoured to distract her thoughts by reading, but it would not do; and at last her father brushed aside the purdah of Mrs. Brent's door, and came out. She cast a questioning look at his anxious face, and he answered it with a single word:

"Cholera."

Kate said nothing; she sank back into her chair again, unable to remove her eyes from her father's face. She could not at once realise that the word he had uttered was a sentence of death; it had stunned her; she could not grasp what it implied.

That was the longest night Kate Harrison had ever passed. She was not allowed to see her aunt, for her own safety; and she spent the interminable hours walking up and down, waiting, helplessly waiting, conscious that nothing could avail to keep off the great messenger who was hovering over the house. The terrible moans of pain ceased soon after midnight, and the silence which followed was unendurable. Dr. Harrison came out, and advised her to go to bed, but in tones which she knew were not meant to enforce obedience; she sat down again to wait, for his manner told her that the end was near.

It came just as the first faint glow of dawn brightened the eastern sky, and called the Indian world to life; came so quietly that the two watchers hardly knew the moment when their vigil was done. They came out of the room together, and gently closed the doors; it was all over: Agnes Brent had gone. The cholera had begun and finished its work in little more than eight hours.

They buried her that evening, with all the hideous gruesome pomp which disfigures poor humanity's last journey upon earth in India. The tawdry emblems of mourning on the shabby hearse, and the native attendants in their unaccustomed garb of black made a melancholy parody of the ceremony which has little to recommend it at home, but Kate scarcely noticed these things, often as she had seen and recoiled from them before. The climate forbids the solemn deliberate preparations for a funeral which is usual in England, and twelve hours after Agnes Brent had been called away she had been laid to rest in the Meerut Cemetery. No beautiful well-tended garden, like the sleeping place of the dead in Europe; a neglected, jungle smothered wilderness, where monuments were falling into decay, untouched since the hands which raised them had departed—some perchance to graves of their own, and others to the ends of the earth. They had cleared a space, and prepared a grave for Mrs. Brent's remains, at a spot selected by her brother, amid a cluster of tiny mounds which marked the last beds of children. There are many of those little graves in an Indian cemetery.

(*To be continued.*)

